# The Mechanisms and Animal Models of SARS-CoV-2 Infection

**DOI:** 10.3389/fcell.2021.578825

**Published:** 2021-04-27

**Authors:** Wenrui Jia, Juan Wang, Bao Sun, Jiecan Zhou, Yamin Shi, Zheng Zhou

**Affiliations:** ^1^Department of Chinese Medicine, The First Affiliated Hospital of Zhengzhou University, Zhengzhou, China; ^2^Department of Pharmacy, The Second Xiangya Hospital, Central South University, Changsha, China; ^3^Institute of Clinical Medicine, The First Affiliated Hospital, University of South China, Hengyang, China

**Keywords:** angiotensin converting enzyme II, animal models, immunopathology, pathogenesis, severe acute respiratory syndrome coronavirus 2

## Abstract

Coronavirus disease 2019 (COVID-19) is a highly contagious disease caused by severe acute respiratory syndrome coronavirus 2 (SARS-CoV-2), which has aroused great public health concern worldwide. Currently, COVID-19 epidemic is spreading in many countries and regions around the world. However, the study of SARS-CoV-2 is still in its infancy, and there is no specific therapeutics. Here, we summarize the genomic characteristics of SARS-CoV-2. In addition, we focus on the mechanisms of SARS-CoV-2 infection, including the roles of angiotensin converting enzyme II (ACE2) in cell entry, COVID-19 susceptibility and COVID-19 symptoms, as well as immunopathology such as antibody responses, lymphocyte dysregulation, and cytokine storm. Finally, we introduce the research progress of animal models of COVID-19, aiming at a better understanding of the pathogenesis of COVID-19 and providing new ideas for the treatment of this contagious disease.

## Introduction

Coronavirus disease 2019 (COVID-19), caused by severe acute respiratory syndrome coronavirus 2 (SARS-CoV-2), is a highly contagious disease (Wu F. et al., 2020; Zhou P. et al., 2020; Zhu N. et al., 2020). Its most common symptoms are fever, cough, and fatigue, while other symptoms include sputum production, headache, gastrointestinal symptoms, liver injury, and even olfactory and gustatory dysfunctions (Guan et al., 2020; Lechien et al., 2020; Lin et al., 2020; Zhang C. et al., 2020). Meanwhile, the typical chest computerized tomography (CT) imaging features are ground glass opacities in bilateral multiple lobular, consolidation, adjacent pleura thickening and combined linear opacities (Xu et al., 2020). Unlike previously infected coronaviruses, SARS-CoV-2 is identified as the seventh member of the coronavirus family that infects humans (Zhu N. et al., 2020). COVID-19 has spread to many countries and regions and was judged as the sixth public health emergency of international concern (PHEIC) by the World Health Organization (WHO) (Eurosurveillance Editorial Team, 2020). As of March 2021, more than 120 million cases of COVID-19 have been reported worldwide, including 2.7 million deaths (European Centre for Disease Prevention and Control, 2021).

The bat was considered to be a probable natural reservoir host of SARS-CoV-2, whose whole genome was highly similar to a bat coronavirus RaTG13, with a genome sequence identity of 96.2% (Zhou P. et al., 2020). Considering that SARS-CoV is transmitted to humans through masked palm civets and middle east respiratory syndrome coronavirus (MERS-CoV) through dromedary camels, it is also possible that SARS-CoV-2 has intermediate hosts that mediate its transmission (Guan et al., 2003; Alagaili et al., 2014). The latest study discovered that multiple lineages of pangolin coronavirus were similar to SARS-CoV-2, suggesting that pangolin might be a potential intermediate host for the new coronavirus (Lam et al., 2020; Zhang T. et al., 2020). Epidemiologically, the prevalence of COVID-19 is high and the population is generally susceptible, but people with chronic underlying diseases such as diabetes, hypertension, and heart disease are more susceptible to this virus (Wang D. et al., 2020). So far, the study of SARS-CoV-2 is still in its infancy. In this paper, we focus on the mechanisms and animal models of SARS-CoV-2 infection, in order to provide a theoretical basis for understanding the pathogenesis of COVID-19 and the prevention and treatment of the disease.

## Genomic Characterization of Sars-CoV-2

Phylogenetic analysis showed that SARS-CoV-2 was a new betacoronavirus belonging to the sarbecovirus subgenus of Coronaviridae family, which had the typical features of betacoronavirus, such as 5′ untranslated region (UTR), replicase complex (orf1ab) gene, Spike (S) gene, Envelope (E) gene, Membrane (M) gene, Nucleocapsid (N) gene, and 3′ UTR (Zhu N. et al., 2020). The nucleotide sequence of the S gene of SARS-CoV-2 was less than 75% identical to the nucleotide sequence of all the previously described SARS-related coronaviruses, except for a 93.1% identity to the bat coronavirus RaTG13 (Zhou P. et al., 2020). Compared to SARS-CoV, the SARS-CoV-2 S gene had three short insertions in the N-terminal domain and changes in four of the key residues in the receptor-binding motif. Furthermore, the SARS-CoV-2 orf8 was distant from the conserved orf8 or orf8b of SARS-CoV, and this new orf8 might encode a protein with an alpha-helix, following with a beta-sheet(s) (Chan and Kok, 2020). During the SARS epidemic, a common genetic change in SARS-CoV genome was the major deletions in the orf8 region (Chinese SARS Molecular Epidemiology Consortium, 2004; Muth and Corman, 2018). Interestingly, orf8 deletion SARS-CoV-2 variants seem to be associated with mild infections (Su and Anderson, 2020; Young et al., 2020a). In mammals, the zinc finger antiviral protein (ZAP) mediated the degradation of the RNA genome by specifically binding to the CpG dinucleotide in the viral RNA genome (Takata et al., 2017). Notably, among all known betacoronavirus, the CpG defect in the SARS-CoV-2 genome was the most severe, indicating that SARS-CoV-2 might have evolved in a host with high ZAP expression (Xia, 2020). Yang and Chen (2020) discovered that the composition of human-specific slow codons and two consecutive slow codons in SARS-CoV-2 and SARS-CoV was lower than that of other coronaviruses, indicating that these two coronaviruses might have faster protein synthesis. In addition, compared with SARS, bat SARS and MERS-CoV, SARS-CoV-2 had higher codon bias and gene expression efficiency (Kandeel et al., 2020). Specifically, most high frequency codons ended in A or T, while low frequency codons ended in G or C. Meanwhile, the effective number of codon (ENc) values of the SARS-CoV-2 structural proteins was 5–20 lower. These unique genomic features provide the basis for understanding the source and pathogenicity of this novel coronavirus.

## Pathogenicity of Sars-CoV-2

### Roles of Angiotensin Converting Enzyme II (ACE2) in SARS-CoV-2 Infection

#### Receptor Interactions and Cell Entry

Coronavirus infection depends on the binding of its own S glycoprotein to cellular receptors and on the priming of S glycoprotein by cellular proteases. Preliminary studies found that SARS-CoV-2 entered host cells through the same receptor angiotensin converting enzyme II (ACE2) as SARS-CoV (Zhou P. et al., 2020). Moreover, VeroE6 cells with high expression of transmembrane protease serine 2 (TMPRSS2) were very susceptible to SARS-CoV-2 infection, suggesting that TMPRSS2 might be a key protease for SARS-CoV-2 cell entry and replication (Matsuyama et al., 2020). Consistently, Hoffmann et al. (2020b) demonstrated that ACE2 mediated the cell entry of SARS-CoV-2, while the serine protease TMPRSS2 was used for S protein priming. Meanwhile, camostat mesylate, a TMPRSS2 inhibitor, could block SARS-CoV-2 infection of lung cells, indicating that TMPRSS2 inhibitors might be a potential strategy for the treatment of COVID-19. Furthermore, TMPRSS2 was highly expressed in secretory cells, ciliated cells and type I alveolar epithelial cells (AT1), and SARS-CoV-2 RNA could be detected in these cells, indicating that SARS-CoV-2 mainly infected the secretory cells, ciliated cells and AT1 in lung epithelium (Schuler et al., 2021). Importantly, the expression of TMPRSS2 in cells increased with aging in mice and humans, which might partly explain the rarity of severe respiratory disease in children. Cryo–electron microscopy showed that the main state of the SARS-CoV-2 S trimer has one of the three receptor-binding domains (RBDs) rotated up in an accessible conformation of the receptor (Wrapp and Wang, 2020). Furthermore, the overall interface between the RBD of S glycoprotein and peptidase domain (PD) of ACE2 mediated mainly through the interactions of polar residues (Yan and Zhang, 2020). Specifically, an extended loop region of the RBD crossed the arch-shaped α1 helix of the PD, and the α2 helix and the loop connecting the β3 and β4 antiparallel chains of the PD also contributed to the coordination of RBD.

Noteworthily, SARS-CoV-2 mainly entered 293/hACE2 cells through endocytosis, and phosphatidylinositol 3-phosphate 5-kinase (PIKfyve) and two pore channel subtype 2 (TPC2) played an important role in this process (Ou et al., 2020). Moreover, cathepsins in lysosome, especially cathepsin L, were involved in the priming of SARS-CoV-2 S protein. In particular, the S glycoprotein of SARS-CoV-2 contained a furin-like cleavage site within the S1/S2 domain, which was potentially involved in the priming of S protein (Wang Q. et al., 2020). Hoffmann et al. (2020a) discovered that furin could cleave the S glycoprotein at the S1/S2 site, and this cleavage promoted the S protein-mediated virus-cell fusion and cell-cell fusion. Collectively, these endogenous molecules such as ACE2, TMPRSS2, and furin were essential for SARS-CoV-2 infection, which could serve as potential targets for the treatment of COVID-19.

#### ACE2 and COVID-19 Susceptibility

Angiotensin converting enzyme II is the receptor for the entry of SARS-CoV-2 into host cells. Therefore, it is easy to think that its high expression may increase the susceptibility to infection. Based on this, the researchers found that many factors, including population, cancer, lupus, smoking, obesity and diabetes, all may increase the risk of COVID-19.

The genetic analysis of ACE2 in different populations showed that the East Asian populations had higher allele frequencies (AFs) of the expression quantitative trait loci (eQTL) variants than European populations, and these variants were associated with high ACE2 expression in tissues, indicating that different populations might have different susceptibility to SARS-CoV-2 (Cao et al., 2020a). Chai et al. (2020) discovered that ACE2 was overexpressed in certain cancers, such as colon adenocarcinoma, rectal adenocarcinoma, gastric adenocarcinoma, kidney renal papillary cell carcinoma, pancreatic adenocarcinoma, and lung adenocarcinoma. Meanwhile, ACE2 in tumors with high ACE2 expression usually showed hypomethylation. These results explain the higher risk of COVID-19 in cancer patients from the perspective of ACE2. Similarly, lupus patients also presented overexpression and hypomethylation of ACE2, suggesting that patients with such autoimmune diseases might have increased sensitivity COVID-19 (Sawalha et al., 2020). Meanwhile, the epigenetic dysregulation of interferon (IFN)-regulated genes in lupus patients might enhance the immune response to SARS-CoV-2, thereby increasing the severity of COVID-19. Of note, ACE2 expression was upregulated in the small airway epithelia of smokers and patients with chronic obstructive pulmonary disease (COPD), which partly explained the increased risk of SARS-CoV-2 infection in these people (Leung et al., 2020). Consistently, mice exposed to cigarette smoke showed a dose-dependent increase in ACE2 expression, while quitting smoking reduced ACE2 levels in cells (Smith et al., 2020). In-depth research found that ACE2 was mainly expressed in a subset of secretory cells of the lung epithelium, and cigarette smoke could increase the number of ACE2+ cells by stimulating the expansion of secretory cells. Interestingly, overweight COPD patients have higher expression levels of ACE2, indicating that obesity is also a risk factor for COVID-19 (Higham and Singh, 2020). Furthermore, a phenome-wide Mendelian randomization (MR) study showed that diabetes and its related traits were associated with increased expression of ACE2 in the lung, which in turn might mediate susceptibility to COVID-19 (Rao et al., 2020).

#### ACE2 and COVID-19 Symptoms

By analyzing the expression levels of ACE2 in 31 normal human tissues, Li M. Y. et al. (2020) found that small intestine, testis, kidney, heart, thyroid, and adipose tissue expressed high levels of ACE2, and lung, colon, liver, bladder, and adrenal gland expressed moderate levels of ACE2, while blood, spleen, bone marrow, brain, blood vessels, and muscle expressed low levels of ACE2. Importantly, ACE2 expression was related to the immune signatures of each tissue. For example, the expression of ACE2 in the lungs of the elderly was positively correlated with immune signatures, while the expression of ACE2 in the lungs of the young was negatively correlated with immune signatures.

Actually, the expression pattern and expression level of ACE2 in different organs and tissues to some extent determine the symptoms and outcome of COVID-19. Lukassen et al. (2020) observed that ACE2 and TMPRSS2 were primarily expressed in alveolar type II (AT2) cells in the lung and in transient secretory cells in subsegmental bronchial branches, suggesting that these cell types might be susceptible to SARS-CoV-2 infection. Electron microscopy showed that both bronchiole epithelial cells and AT2 cells showed obvious coronavirus particles, and immunohistochemical staining showed the presence of SARS-CoV-2 nucleocapsid in lung tissue, confirming lung injury secondary to SARS-CoV-2 infection (Yao et al., 2020). Usually, COVID-19 patients had some gastrointestinal symptoms, such as nausea, vomiting, and diarrhea (Jin et al., 2020; Song et al., 2020). ACE2 was highly expressed in enterocytes, and SARS-CoV-2 effectively infected enterocytes in human small intestinal organoids (hSIOs), indicating that the intestine might be a target organ of the new coronavirus (Lamers et al., 2020). Interestingly, although the expression levels of ACE2 in enterocytes were much higher than that in enterocyte-precursors, the infection rates of both were similar, suggesting that low ACE2 levels might be sufficient for SARS-CoV-2 to invade host cells. Consistently, Zhou J. et al. (2020) found that SARS-CoV-2 not only actively replicated in the human intestinal organoids, but also could infect bat intestinal cells. At the same time, they isolated infectious SARS-CoV-2 from the stool of a diarrheal COVID-19 patient. These findings suggest that the intestine may be a transmission route of SARS-CoV-2, which may lead to local and overall disease progression. Furthermore, acute kidney injury was one of the important complications of COVID-19 (Huang et al., 2020; Lescure et al., 2020). It was reported that there were coronavirus particles with distinctive spikes in the tubular epithelium and podocytes, and immunostaining with SARS-CoV nucleoprotein antibody was positive in tubules, which provided a basis for SARS-CoV-2 to directly infect kidney tissue (Su et al., 2020). Single-cell transcriptome analysis found that ACE2 and TMPRSSs were co-expressed in podocytes and proximal straight tubule cells, which might be candidate kidney host cells (Pan et al., 2020).

**FIGURE 1:**
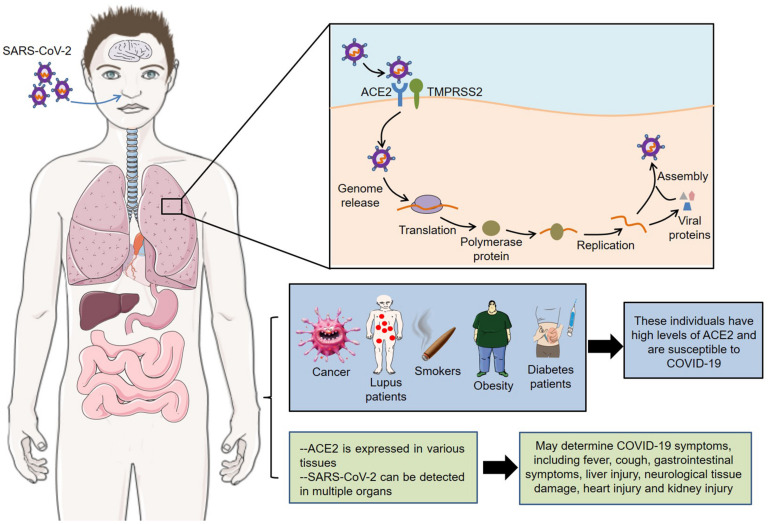
Roles of ACE2 in SARS-CoV-2 infection. Receptor ACE2 mediates the entry of SARS-CoV-2 into host cells. Meanwhile, its expression pattern and level are closely related to the susceptibility and symptoms of COVID-19. SARS-CoV-2, severe acute respiratory syndrome coronavirus 2; ACE2, angiotensin converting enzyme II; COVID-19, coronavirus disease 2019; TMPRSS2, transmembrane protease serine 2.

The latest autopsy findings showed that SARS-CoV-2 could be detected in multiple organs, such as heart, liver, brain, lungs, pharynx, and kidneys (Puelles et al., 2020). In addition, neurological tissue damage, pancreatic damage, heart injury and olfactory dysfunctions might also be related to ACE2 expression pattern in the corresponding tissues (Baig et al., 2020; Bilinska et al., 2020; Chen L. et al., 2020; Liu F. et al., 2020). However, these data are mainly from different tissues from healthy human donors, and more animal and cell experiments are needed to verify the pathological mechanism of COVID-19 symptoms. In short, receptor ACE2 mediates the entry of SARS-CoV-2 into host cells, and its expression pattern and level also play important roles in COVID-19 susceptibility and symptoms (Figure 1).

### Immunopathology of COVID-19

**FIGURE 2:**
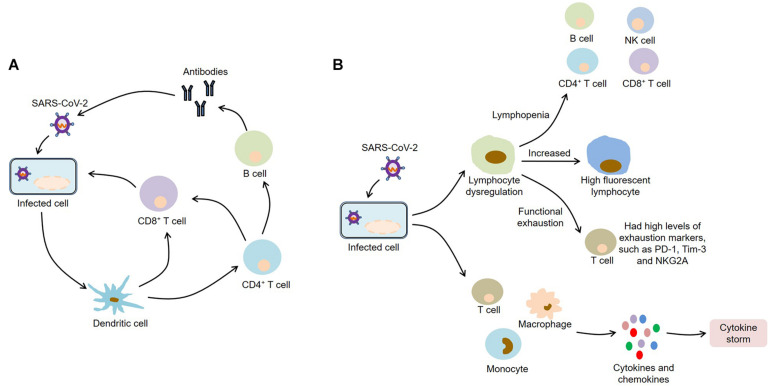
Immunopathology of COVID-19. **(A)** SARS-CoV-2 infection activates humoral and cellular immunity mediated by virus-specific B and T cells; **(B)** SARS-CoV-2 infection causes lymphocyte dysregulation and cytokine storm, which contribute to the development of COVID-19. SARS-CoV-2, severe acute respiratory syndrome coronavirus 2; COVID-19, coronavirus disease 2019; NK, natural killer; PD-1, programmed death-1; Tim-3, T-cell immunoglobulin and mucin-domain containing-3; NKG2A, CD94/NK group 2 member A.

After infecting with SARS-CoV-2, macrophages and monocytes are recruited and subsequently release cytokines, thereby activating humoral and cellular immunity mediated by virus-specific B and T cells. However, impaired adaptive immune response and uncontrolled systemic inflammatory response may cause severe organ damage and even death (Figure 2).

#### Antibody Responses

Serum from COVID-19 patients cross-reacted with the nucleocapsid antigens of SARS-CoV, but showed no cross-binding with the S1 subunit of SARS-CoV peak antigen (Long et al., 2020). Within 19 days after symptom onset, the proportion of virus-specific immunoglobulin-G (IgG)-positive patients reached 100%, and within 22 days after symptom onset, the proportion of virus-specific IgM-positive patients reached a maximum of 94.1%. Moreover, IgM seroconversion increased rapidly from the 9th day after the onset of symptoms, while IgG seroconversion increased from the 11th day after the onset of symptoms (Xiang et al., 2020). Importantly, both specific IgM and IgG antibodies showed obvious specificity and positive predictive value, suggesting that antibody detection might help the diagnosis of COVID-19. Pediatric patients with COVID-19 could develop a protective humoral immunity (Zhang Y. et al., 2020). Specifically, compared with the uninfected controls, the percentage of IgG+ B cells were slightly higher in total B cells in infected children, while the percentage of IgG+ B cells in memory B cells was significantly higher. Meanwhile, total and IgG antibodies against the nucleocapsid protein and spike-RBD of SARS-CoV-2 were found in infected children around 2–3 weeks after the onset. Additionally, Wen et al. (2020) demonstrated that some new changes of B cell receptor (BCR) in the recovery stage of COVID-19 patients, especially IGHV3-23 and IGHV3-7, provided new ideas for the design of vaccines. Notably, Zeng F. et al. (2020) found that the mild and recovering patients with COVID-19 had no difference in the concentration of specific IgG antibody between sexes, while in severe conditions, there were higher serum IgG antibody concentrations in female patients, which might be related to the different outcomes between male and female COVID-19 patients.

Clinically, convalescent plasma has been used to treat critically ill patients with SARS-CoV-2 infection, which improved the clinical outcomes and showed good tolerance (Duan et al., 2020; Shen et al., 2020). At the same time, scientists are working to develop monoclonal antibodies that neutralize SARS-CoV-2. Wrapp et al. (2020) isolated two single-domain antibodies (VHHs), MERS VHH-55 and SARS VHH-72, from a llama immunized with prefusion-stabilized MERS-CoV and SARS-CoV S proteins. These VHHs could bind to the RBDs of the S proteins of MERS-CoV and SARS-CoV, and neutralize S pseudotyped viruses *in vitro*. Strikingly, the SARS VHH-72 cross-reacted with the SARS-CoV-2 RBD, and its bivalent IgG Fc-fusion VHH-72-Fc neutralized SARS-CoV-2 S pseudovirus, suggesting that VHH-72-Fc might act as a potential therapeutic candidate for COVID-19. CR3022, a neutralizing antibody isolated from a SARS patient, could effectively bind to the RBD of SARS-CoV-2 by recognizing an epitope that did not overlap with the ACE2 binding site (Tian et al., 2020). Further studies discovered that CR3022 targeted a conserved epitope distal from the receptor binding site, and the binding could only occur when at least two RBDs in the S trimer were in the up conformation and slightly rotated (Yuan and Wu, 2020). Unfortunately, CR3022 did not neutralize SARS-CoV-2 *in vitro*. Another research also found that cross-reactive binding to RBD and non-RBD regions was common between SARS-CoV and SARS-CoV-2, while the cross-neutralization of these two live viruses might be rare (Lv et al., 2020).

Of note, Chen X. et al. (2020) found that recovered COVID-19 patients produced the antibodies against S1 subunit, but only a small part could block the binding of RBD to ACE2 receptor. Subsequently, they cloned two monoclonal antibodies, 311mab-31B5 and 311mab-32D4, by using SARS-CoV-2 RBD-specific memory B cells isolated from recovered COVID-19 patients, which could block the binding of SARS-CoV-2 RBD to ACE2 receptor and effectively neutralize SARS-CoV-2 S protein pseudovirus. Similarly, Cao et al. (2020b) identified a neutralizing antibody BD-368-2 with strong ability to neutralize pseudotyped SARS-CoV-2 by high-throughput sequencing of antigen-binding single B cells, which could effectively treat and prevent SARS-CoV-2 infection in hACE2 transgenic mice. In fact, several cross-neutralizing antibodies, including COVA1-16, S309, H014, 47D11, ADI-56046, and 515-5, have been discovered, which provide a theoretical basis for the development of SARS-CoV-2 vaccines and therapies (Liu H. et al., 2020; Lv and Deng, 2020; Pinto et al., 2020; Wan et al., 2020; Wang and Li, 2020; Wec and Wrapp, 2020). COVA1-16 bound to SARS-CoV-2 RBD by a long complementarity-determining region H3, which blocked the interaction between ACE2 and RBD through steric hindrance rather than epitope overlap (Liu H. et al., 2020). Another human monoclonal antibody S309 were found from the memory B cells of SARS patients, which could not only neutralize SARS-CoV and SARS-CoV-2 pseudoviruses, but also neutralize live SARS-CoV-2 by binding to the RBD of the S glycoprotein (Pinto et al., 2020). It was worth noting that when other monoclonal antibodies were used in combination with S309, S309 further enhanced their neutralization, indicating that the antibody cocktails containing S309 might be an effective method for the treatment of COVID-19. Moreover, 47D11 could neutralize SARS-CoV and SARS-CoV-2 in cell culture (Wang and Li, 2020). Although this monoclonal antibody targeted a conserved epitope on the RBD of S protein, it did not compete with the S protein for interaction with the ACE2 receptor, suggesting that 47D11 blocked SARS-CoV-2 infection by a mechanism different from receptor binding-inhibition. In conclusion, these monoclonal antibodies may be an effective means of preventing and treating COVID-19.

#### Lymphocyte Dysregulation

Lymphocytes, such as B cells, CD4^+^ T cells, CD8^+^ T cells, and natural killer (NK) cells, are important components of the immune system. Strikingly, most COVID-19 patients usually show significant lymphopenia (Young et al., 2020b; Zhang J. J. et al., 2020). Specifically, total lymphocytes, B cells, CD4^+^ T cells, CD8^+^ T cells, and NK cells were decreased in COVID-19 patients, and severe patients were more severe than mild patients (Wang F. et al., 2020). Meanwhile, the CD8^+^ T cells and CD4^+^/CD8^+^ ratio were significantly related to the inflammation status of COVID-19, while the decrease in CD8^+^ T cells and B cells and the increase in CD4^+^/CD8^+^ ratio were associated with poor efficacy after treatment, indicating that some peripheral lymphocyte subsets could be used as predictors of disease progression and treatment effect. Zhang and Tan (2020) confirmed that the decrease in lymphocyte counts in severe patients was negatively correlated with serum levels of interleukin-6 (IL-6) and IL-8. Moreover, the neutrophil to lymphocyte ratio (NLR) was often higher in severe COVID-19 patients and was an independent risk factor for mortality in hospitalized patients (Liu Y. et al., 2020). Notably, the reduced B cells were related to the prolongation of viral RNA shedding from respiratory tract in COVID-19 patients (Hao et al., 2020). Interestingly, high fluorescent lymphocytes (HFL) associated with activated B cells or plasma cells were elevated in COVID-19 patients (Wang Z. et al., 2020). HFL tended to increase as the disease progressed, which might promote the production of plasma cells and specific antibodies in patients. Ganji et al. (2020) found that the ratio of CD4 to CD8 was not significantly different between COVID-19 patients and healthy controls. However, COVID-19 patients had higher levels of CD8 expression, indicating that host T lymphocytes might increase their cytotoxic activity by upregulating CD8 during SARS-CoV-2 infection, thereby exerting antiviral effect.

In addition, the functional state of T cells in COVID-19 patients is also dysregulated. Compared with the healthy controls, T cells from COVID-19 patients had higher levels of the exhaustion markers, including programmed death-1 (PD-1) and T-cell immunoglobulin and mucin-domain containing-3 (Tim-3) (Diao et al., 2020). In the early stage of the disease, the proportions of PD-1^+^ and Tim-3^+^ on CD8^+^ and CD4^+^ T cells were low. As the disease progressed, the expression of PD-1 and Tim-3 in CD8^+^ T cells has a tendency to increase, while the expression of Tim-3 was increased in CD4^+^ T cells, but there is no significant change in PD-1 expression. These results indicate that T cells from COVID-19 patients have an exhaustion phenotype. Consistently, another study found that the levels of tumor necrosis factor-α (TNF-α) and IFN-γ in CD4^+^ T cells of severe COVID-19 patients were lower than those of mild patients, while the levels of granzyme B and perforin in CD8^+^ T cells of severe patients were higher than those of mild patients (Zheng H. Y. et al., 2020). Meanwhile, the levels of human leukocyte antigen D related (HLA-DR) and T cell Ig and ITIM domain (TIGIT) in CD8^+^ T cells of severe patients were higher than those of mild patients. These findings suggest that SARS-CoV-2 infection can affect the functions of CD4^+^ T cells and promote the excessive activation and exhaustion of CD8^+^ T cells. Furthermore, the expression of CD94/NK group 2 member A (NKG2A) was increased on cytotoxic T lymphocytes (CTLs) and NK cells in COVID-19 patients, accompanied by low levels of CD107a, IFN-γ, IL-2, TNF-α, and granzyme B (Zheng M. et al., 2020). Importantly, effectively controlled COVID-19 patients had increased levels of NK cells and CD8^+^ T cells, as well as decreased NKG2A expression, indicating that targeting NKG2A might prevent functional exhaustion of lymphocytes and thus contribute to COVID-19 recovery.

Actually, SARS-CoV-2 infected T lymphocytes through membrane fusion mediated by S protein, which could be inhibited by EK1 peptide (Wang X. et al., 2020). However, SARS-CoV-2 could not replicate in MT-2 cells. Furthermore, CD147 was another receptor that mediated SARS-CoV-2 infection (Ulrich and Pillat, 2020). Because CD147 existed on the surface of T lymphocytes, it might promote the SARS-CoV-2 infection of T cells. By transcriptome sequencing of the bronchoalveolar lavage fluid (BALF) and peripheral blood mononuclear cells (PBMCs) of COVID-19 patients, Xiong et al. (2020) found that some significantly altered genes were enriched in apoptosis and P53 pathway. These results may partially explain the cause of lymphopenia in patients with COVID-19.

#### Cytokine Storm

In response to infections, large amounts of cytokines released by immune cells may cause cytokine storm or macrophage activation syndrome (MAS), which is an excessive inflammation response to external stimuli. In this scenario, the immune system attacks normal organs and tissues, leading to acute respiratory distress syndrome (ARDS), multiple organ failure, and even death (Ye et al., 2020). Most patients with severe COVID-19 exhibited large amounts of pro-inflammatory cytokines and chemokines, such as IL-2, IL-6, IL-7, TNF-α, granulocyte colony stimulating factor (GCSF), CXC-chemokine ligand 10 (CXCL10), CC-chemokine ligand 2 (CCL2), and CCL3 (Chen N. et al., 2020; Huang et al., 2020). Consistently, the metatranscriptomic sequencing of the BALF discovered that COVID-19 patients showed marked hypercytokinemia and up-regulated IFN-stimulated genes, especially significantly elevated expression of chemokines, such as CXCL17, CXCL8, CCL2, and CCL7 (Zhou Z. et al., 2020). Interestingly, most cytokines were expressed after respiratory function nadir, whereas only IL-1 was induced before respiratory function nadir (Ong et al., 2020). Furthermore, inflammatory markers in blood, including C-reactive protein (CRP), ferritin, and D-dimer, were found to be abnormally up-regulated in patients (Tan et al., 2020; Zhou F. et al., 2020). Importantly, these cytokines and chemokines have been associated with disease severity and death. For example, IL-6, CRP, and hypertension were ideal predictors for the progression of COVID-19 (Zhu Z. et al., 2020), while older age, high Sequential Organ Failure Assessment (SOFA) score and D-dimer greater than 1 μg/L were risk factors for in-hospital death in COVID-19 patients (Zhou F. et al., 2020). Therefore, strategies to suppress inflammatory responses are particularly important in patients with COVID-19, especially in severe cases.

## Animal Models of COVID-19

**FIGURE 3:**
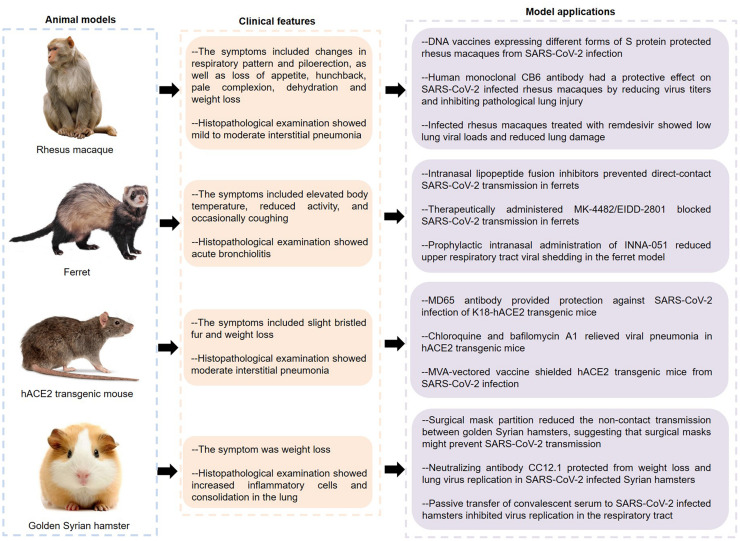
Features and applications of animal models of COVID-19. SARS-CoV-2, severe acute respiratory syndrome coronavirus 2; hACE2, human angiotensin converting enzyme II; COVID-19, coronavirus disease 2019.

As can be seen from the above, current studies on COVID-19 pathogenesis are mainly focused on ACE2 and immunopathology. It is of great significance to further explore the molecular mechanisms. In this regard, effective animal models of COVID-19 are indispensable. So far, SARS-CoV-2 infection has been described in various animal models such as macaque, ferret, mouse, and hamster (Figure 3).

### Non-human Primates, Including Cynomolgus Macaque and Rhesus Macaque

Rockx and Kuiken (2020) inoculated a SARS-CoV-2 strain into young and aged cynomolgus macaques by a combination of intratracheal and intranasal routes, causing a COVID-19-like disease. Specifically, none of these macaques showed obvious clinical symptoms and weight loss, except for one elderly who developed a serous nasal discharge on the 14th day after inoculation. The virus was mainly expelled from the nose and throat, and peaked early in the infection. Of note, one macaque showed shedding of viral RNA in rectal swabs on the 14th day. The autopsy of four macaques showed that SARS-CoV-2 RNA was detected in multiple tissues from the respiratory tract, ileum and tracheobronchial lymph nodes. Importantly, two out of four macaques had areas with acute or more advanced diffuse alveolar damage (DAD), whose histological features including alveolar edema, epithelial necrosis, hyaline membrane formation, and accumulation of neutrophils, macrophages, and lymphocytes.

Analogously, rhesus macaques inoculated with SARS-CoV-2 had a respiratory disease (Munster et al., 2020). The symptoms of infected animals included changes in respiratory pattern and piloerection, as well as loss of appetite, hunchback, pale complexion, dehydration and weight loss, and lung radiographs showed pulmonary infiltrates. Histologically, 3 out of 4 animals developed mild to moderate interstitial pneumonia with the features of thickening of alveolar septa, alveolar edema and hyaline membranes, indicating that the macaque model basically recapitulates the pathological features of COVID-19. Interestingly, Chandrashekar and Liu (2020) found that the rhesus macaques infected with SARS-CoV-2 were difficult to re-infect this virus, and the macaques showed anamnestic immune responses following rechallenge, indicating that protective immunity might play a role in this process. Furthermore, rhesus macaques vaccinated with DNA vaccines expressing different forms of S protein produced humoral and cellular immune responses (Yu and Tostanoski, 2020). Importantly, when the vaccinated animals were infected with SARS-CoV-2, their bronchoalveolar lavage and nasal mucosa showed reduced viral loads, indicating that these vaccines had protective effects on non-human primates. Notably, CB6, a human monoclonal antibody, had a protective effect on SARS-CoV-2 infected rhesus macaques by reducing virus titers and inhibiting pathological lung injury, indicating that CB6 might be a potential treatment for COVID-19 (Shi and Shan, 2020). Similarly, infected rhesus macaques treated with remdesivir showed low lung viral loads and reduced lung damage, which provided a basis for the clinical application of remdesivir (Williamson et al., 2020).

### Ferret

Strikingly, ferrets were highly sensitive to SARS-CoV-2 infection and could transmit the virus to other ferrets in direct or indirect contact (Kim et al., 2020). The infected ferrets had elevated body temperature, reduced activity, and occasionally coughing. Meanwhile, viral RNA was detected in nasal turbinate, lung, trachea, intestine, and kidney tissues. Importantly, infected ferrets had more immune infiltration and cellular debris in the bronchial epithelium, bronchial lumen and alveolar wall, indicating that SARS-CoV-2 infection could cause acute bronchiolitis in ferrets. Additionally, many up-regulated cytokines and chemokines, such as IL-6, CCL2, CCL8, and CXCL9, were found in the nasal washes of ferrets infected with SARS-CoV-2, indicating that this animal model also showed an significant inflammatory response (Blanco-Melo et al., 2020). Interestingly, the newly designed lipoprotein fusion inhibitors inhibited the conformational changes of S protein and blocked the membrane fusion between the virus and host cell membrane by integrating into the host cell membranes, and their intranasal administration could effectively inhibit SARS-CoV-2 transmission in ferrets (de Vries and Schmitz, 2021). Similarly, the ribonucleoside analog inhibitor MK-4482/EIDD-2801 not only reduced the viral load in the upper respiratory tract, but also inhibited the viral transmission in ferrets, indicating that MK-4482/EIDD-2801 might become a potential drug to control the transmission of SARS-CoV-2 in the community (Cox et al., 2021). Recent studies found that preventive intranasal administration of TLR2/6 agonist INNA-051 could effectively reduce the virus levels in throat swabs and nasal washes of the ferrets infected with SARS-CoV-2 (Proud et al., 2021). Of note, although lopinavir-ritonavir, emtricitabine-tenofovir, and hydroxychloroquine sulfate slightly alleviated the clinical symptoms of SARS-CoV-2 infected ferrets, they did not significantly reduce the virus titers (Park et al., 2020). Therefore, more clinical studies are needed to evaluate the effects of these drugs on COVID-19.

### Mouse

Bao et al. (2020a) generated hACE2 transgenic mice through microinjecting the mice ACE2 promoter driving the hACE2 coding sequence into the fertilized ova. Transgenic mice infected with SARS-CoV-2 showed slight bristled fur and weight loss. The lung tissues of infected mice showed moderate interstitial pneumonia characterized by thickened alveolar septa, accumulation of inflammatory cells in alveolar cavities, and fragmentation of bronchiolar epithelial cells. Importantly, the co-localization of ACE2 and S protein was found in alveolar epithelial cells. Similar to ferrets, SARS-CoV-2 could be spread in hACE2 transgenic mice through close contact and respiratory droplets (Bao et al., 2020b). These results indicate that the hACE2 transgenic mice can serve as valuable animal models for SARS-CoV-2 infection. Human monoclonal MD65 antibody showed high neutralization ability to SARS-CoV-2 *in vitro*, and it had a significant protective effect on SARS-CoV-2 infected K18-hACE2 transgenic mice (Rosenfeld and Noy-Porat, 2021). This protective effect is manifested in the improvement of lung inflammation and pathological processes, as well as the life-saving of infected mice. Furthermore, some endosomal acidification inhibitors, such as chloroquine, NH4CL, and bafilomycin A1, have been confirmed to have antiviral effects in SARS-CoV-2 infected cell or animal models (Shang et al., 2021). Among them, the lungs of infected mice treated with chloroquine and bafilomycin A1 showed reduced infiltration of inflammatory cells and improved alveolar structure. Liu et al. (2021) demonstrated that the recombinant modified vaccinia virus Ankaras (rMVAs) expressing modified forms of SARS-CoV-2 S proteins could not only suppress viral replication in the upper and lower respiratory tracts, but also reduce the expression of cytokines and chemokines in the lungs of hACE2 mice, suggesting that this vaccine had the potential to control COVID-19 during the current pandemic.

Besides hACE2 transgenic mice, hACE2 mice established by CRISPR/Cas9 knock-in technology, adeno-associated virus encoding for hACE2 (AAV-hACE2)-transduced mice, replication-deficient adenovirus encoding hACE2 (Ad5-hACE2)-transduced mice and wild-type mice infected with mouse-adapted SARS-CoV-2 have also been used in the pathological study of COVID-19 (Dinnon et al., 2020; Israelow et al., 2020; Sun J. et al., 2020; Sun S. H. et al., 2020). hACE2 mice established by CRISPR/Cas9 developed interstitial pneumonia with an increase in various cytokines after SARS-CoV-2 infection (Sun S. H. et al., 2020). Moreover, inoculation of SARS-CoV-2 in the stomach could cause a productive infection in hACE2 mice, indicating that SARS-CoV-2 might be transmitted through the fecal-oral route (Sun S. H. et al., 2020). Similarly, Ad5-hACE2-transduced mice showed some symptoms of pneumonia, and treatment with remdesivir or human convalescent plasma 1 day prior to infection could accelerate virus clearance and prevent weight loss and lung histological changes in mice, suggesting that this mouse model was very suitable for the evaluation of COVID-19 therapies (Sun J. et al., 2020). The SARS-CoV-2 variant containing the N501Y mutation could bind to mouse ACE2, causing wild-type mice to be infected with SARS-CoV-2 and develop interstitial pneumonia (Gu and Chen, 2020). Because the cost and practicality of this model are superior to other animal models, it has received great attention.

### Golden Syrian Hamsters

In addition, SARS-CoV-2 could be highly transmitted among golden Syrian hamsters via direct contact and aerosols, and infected hamsters usually showed weight loss and increased inflammatory cells and consolidation in the lungs (Sia et al., 2020). The researchers discovered viral antigen in the bronchial epithelial cells, nasal epithelial cells and duodenum epithelial cells. At the same time, viral antigen was also detected in the olfactory sensory neurons at the nasal mucosa, indicating that SARS-CoV-2 might have infected neurons and subsequently caused olfactory dysfunctions. Meaningfully, surgical mask partition could significantly reduce the non-contact transmission between golden Syrian hamsters, and the infected naïve hamsters showed milder clinical symptoms and histopathological changes, suggesting that surgical masks might play an important role in preventing SARS-CoV-2 transmission (Chan et al., 2020). Rogers and Zhao (2020) isolated a variety of neutralizing antibodies from recovered SARS-CoV-2 patients, which recognized the RBD or non-RBD epitopes of the S protein. Of these, the antibody CC12.1 targeting the RBD-A epitope could protect from weight loss and lung virus replication in SARS-CoV-2 infected Syrian hamsters, indicating that this monoclonal antibody had the potential to prevent and treat COVID-19. Moreover, passive transfer of convalescent serum from infected animals to SARS-CoV-2 infected hamsters could efficiently inhibit virus replication in the respiratory tract, indicating that convalescent plasma might serve as a potential therapy for COVID-19 (Imai et al., 2020). In addition, it was reported that certain animals such as dogs, cats and tigers could also be infected with SARS-CoV-2, which not only contributed to understanding the transmission of the virus, but also provided more possibilities for modeling (Halfmann et al., 2020; Sit et al., 2020; Wang L. et al., 2020).

## Conclusion and Future Perspective

All in all, the receptor ACE2 mediates the entry of SARS-CoV-2 into host cells, and its expression pattern and level are closely related to COVID-19 susceptibility and symptoms. Meanwhile, the knowledge of immunopathology related to COVID-19, such as antibody responses, lymphocyte dysregulation, and cytokine storms, is also crucial for understanding SARS-CoV-2 infection. Furthermore, COVID-19 animal models, including macaque, ferret, hamster and hACE2 transgenic mouse, not only help to explore the pathogenesis of COVID-19, but also provide useful tools for evaluating vaccines and therapeutics. However, some problems need to be solved in the future.

Firstly, since SARS-CoV-2 has obvious human-to-human transmission, it is of great significance to understand the transmission route for the prevention and control of the epidemic (Ghinai et al., 2020; Li Q. et al., 2020). Indeed, respiratory transmission is the main mode for SARS-CoV-2, while fecal-oral transmission, vertical maternal-fetal transmission, and transmission through the ocular surface are also potential transmission routes (Wu Y. et al., 2020; Zeng L. et al., 2020; Zhang X. et al., 2020). Secondly, there is no specific therapeutics for COVID-19. Although remdesivir and chloroquine could effectively inhibit SARS-CoV-2 *in vitro*, it was necessary to prove their effectiveness and safety through clinical trials (Wang M. et al., 2020). Interestingly, the cellular nanosponges composed of the plasma membranes of human lung epithelial type II cells or macrophages prevented virus infection by neutralizing SARS-CoV-2 (Zhang Q. et al., 2020). Furthermore, human recombinant soluble ACE2 (hrsACE2) could significantly inhibit the SARS-CoV-2 infection of human organoids, which might be a promising therapeutic (Monteil et al., 2020). Importantly, further research on molecular mechanisms is needed to provide new ideas for the prevention and treatment of COVID-19.

As the number of COVID-19 cases continues to increase globally, effective prevention and control measures are crucial. In addition to building a community prevention and control system to control the spread of the epidemic, achieving herd immunity through vaccination is also an indispensable means. Fortunately, nine vaccines against SARS-CoV-2 have been approved for use in humans, and more than 300 million people have been vaccinated worldwide. However, some complex situations, such as highly infectious SARS-CoV-2 variants, the evasion of viral variants from vaccine-induced humoral immunity, and the limited durability of humoral responses in coronavirus infections, pose severe challenges to the control of COVID-19 (Kaneko et al., 2020; Garcia-Beltran et al., 2021; Zhou and Thao, 2021). In this context, it is of great significance to develop broadly neutralizing antibodies and vaccine boosters that target these SARS-CoV-2 variants.

## Author Contributions

WJ and JW contributed toward the concept and manuscript writing. BS and JZ participated in the literature search and discussion. YS and ZZ revised and supervised overall project. All authors read and approved the final version of manuscript.

## Conflict of Interest

The authors declare that the research was conducted in the absence of any commercial or financial relationships that could be construed as a potential conflict of interest.
